# Abnormal Mitochondrial L-Arginine Transport Contributes to the Pathogenesis of Heart Failure and Rexoygenation Injury

**DOI:** 10.1371/journal.pone.0104643

**Published:** 2014-08-11

**Authors:** David Williams, Kylie M. Venardos, Melissa Byrne, Mandar Joshi, Duncan Horlock, Nicholas T. Lam, Paul Gregorevic, Sean L. McGee, David M. Kaye

**Affiliations:** 1 Heart Failure Research Group, Baker IDI Heart & Diabetes Institute, Melbourne, Australia; 2 Department of Medicine, Monash University, Melbourne, Australia; 3 Muscle Research & Therapeutics Laboratory, Baker IDI Heart & Diabetes Institute, Melbourne, Australia; 4 Metabolic Research Unit, Deakin University, Geelong, Australia; Rutgers New Jersey Medical School, United States of America

## Abstract

**Background:**

Impaired mitochondrial function is fundamental feature of heart failure (HF) and myocardial ischemia. In addition to the effects of heightened oxidative stress, altered nitric oxide (NO) metabolism, generated by a mitochondrial NO synthase, has also been proposed to impact upon mitochondrial function. However, the mechanism responsible for arginine transport into mitochondria and the effect of HF on such a process is unknown. We therefore aimed to characterize mitochondrial L-arginine transport and to investigate the hypothesis that impaired mitochondrial L-arginine transport plays a key role in the pathogenesis of heart failure and myocardial injury.

**Methods and Results:**

In mitochondria isolated from failing hearts (sheep rapid pacing model and mouse Mst1 transgenic model) we demonstrated a marked reduction in L-arginine uptake (p<0.05 and p<0.01 respectively) and expression of the principal L-arginine transporter, CAT-1 (p<0.001, p<0.01) compared to controls. This was accompanied by significantly lower NO production and higher 3-nitrotyrosine levels (both p<0.05). The role of mitochondrial L-arginine transport in modulating cardiac stress responses was examined in cardiomyocytes with mitochondrial specific overexpression of CAT-1 (mtCAT1) exposed to hypoxia-reoxygenation stress. mtCAT1 cardiomyocytes had significantly improved mitochondrial membrane potential, respiration and ATP turnover together with significantly decreased reactive oxygen species production and cell death following mitochondrial stress.

**Conclusion:**

These data provide new insights into the role of L-arginine transport in mitochondrial biology and cardiovascular disease. Augmentation of mitochondrial L-arginine availability may be a novel therapeutic strategy for myocardial disorders involving mitochondrial stress such as heart failure and reperfusion injury.

## Introduction

Heightened oxidative stress is a key feature of numerous chronic diseases including cardiovascular disease and neurodegenerative diseases. In particular the generation of reactive oxygen species (ROS) and reactive nitrogen species (RNS) is now considered to be a central contributor to the development and progression of many diseases, via a range of effects including the oxidative and nitrosative damage of proteins, enzymes, lipids, DNA and cellular membranes [1]. Within the heart, elevated oxidative stress has been reported to play a key role in the pathogenesis of heart failure and ischemia reperfusion injury [2]–[4]. Several sources of cellular ROS production are recognized in the myocardium including the mitochondrial electron transport chain, NADPH oxidase, xanthine oxidase and cytoplasmic NO synthase (NOS) [1], however the relative contribution of each source to overall cellular ROS production has not been clearly defined.

Mitochondrial dysfunction is increasingly understood to be central to the pathophysiology of heart failure and ischemia-reperfusion injury [5], [6]. Recent data indicate that oxidative inhibition of complex I or III of the mitochondrial transport can further increase ROS production most likely due to post-translational modifications [7]. In this context, it has been shown that NO may exert a cardioprotective effect by mitigating oxidative damage of components of the electron transport chain by site-specific S-nitrosation [7]–[9].

Whilst the mechanism underpinning the protective effect of NO within mitochondria is emerging, it remains controversial as to whether NO is derived from mitochondrial or non-mitochondrial sources. In this regard, the presence of a mitochondrial NOS has been reported by several groups as recently reviewed [10]. In this context, some investigators have estimated that a mitochondrial NOS may contribute up to 56% of total cellular production of NO in the heart [11].

Notwithstanding the specific characterization of a mitochondrial NOS, it has been demonstrated that the generation of NO by mitochondria is influenced by the concentration of L-arginine [12]. In this context, it is unclear how L-arginine enters mitochondria and whether this process is altered in cardiovascular disease. Plasmalemmal arginine transport is predominantly mediated by the system y^+^ carrier, which is characterized by sodium independent transport of the cationic amino acids. At normal physiological concentrations of L-arginine the cationic amino acid transporter type 1, CAT-1, is the predominant cellular transport system [13]. Previously, we have shown that CAT-1 mediated L-arginine transport is reduced in the setting of heart failure and hypoxia-reperfusion [14]–[16]. In the current study we aimed to characterize the presence and activity of CAT-1 in cardiac mitochondria and to determine whether altered mitochondrial L-arginine transport contributes to the pathophysiology of heart failure and ischemia reperfusion injury.

## Materials and Methods

The research projects were approved by the Department of Primary Industry Animal Ethics Committee, and the Alfred Medical Research and Education Precinct Animal Ethics Committee. The investigations conform to the NHMRC Australian code of practice for the care and use of animals for scientific purpose, and the Guide for the Care and Use of Laboratory Animals published by the National Institutes of Health.

### Animal Models of Heart Failure and Isolation of Cardiac Mitochondria

Two animal models of heart failure were employed in the present study. Heart failure was induced in adult sheep by rapid ventricular pacing or tachycardia at 190 beats per minute for four weeks as previously described [17]. In brief, pacemakers were implanted under anesthesia using propofol and isoflurane. At the end of the pacing period, animals were killed by pentobarbitone overdose (100 mg/kg) and hearts were immediately excised and washed in ice-cold PBS prior to mitochondrial isolation. In addition to the sheep model, 16 week old transgenic mice with dilated cardiomyopathy due to cardiac specific overexpression of Mst1 [18] (kindly provided by Dr Julie McMullen, Baker IDI) and littermate controls were also investigated in this study. Mice were killed by pentobarbitone overdose (150 mg/kg i.p.).

### Mitochondrial Isolation and Purity

Following cardiac excision, hearts were immediately immersed in ice cold isolation buffer (Mannitol 200 mM, Sucrose 50 mM, KH2PO4 5 mM, EGTA 1 mM, MOPS 5 mM, BSA 0.1%, pH 7.2). For sheep studies, transmural samples of the left ventricle were obtained from the mid-anterior wall. Cardiac tissue was thoroughly minced into ∼1 mm pieces and rinsed with isolation buffer. Tissue pieces were transferred to an ice-cold glass homogeniser vessel containing isolation buffer and 1 mg/ml protease (Sigma) and homogenised for 30 seconds using a Teflon homogeniser pestle [19], [20]. The tissue homogenate was subsequently transferred to an ice-cold 50 mL homogeniser vessel and diluted 10-fold and homogenised for a further 30 seconds. The tissue homogenate was then centrifuged at 8000 g for 10-minutes. The supernatant was discarded and the pellet was carefully re-suspended and then centrifuged at 700 g for 10 minutes. The supernatant containing the mitochondrial fraction was collected and transferred into fresh isolation buffer without EGTA or BSA and centrifuged at 8000 g for 10 min and the resulting pellet was re-suspended in ice cold isolation buffer without EGTA/BSA and kept on ice until required.

Mitochondrial purity was confirmed by western blotting for a mitochondrial specific protein, 70 kDa subunit of Complex II, together with demonstration of exclusion of the plasmalemmal Na^+^K^+^ATPase (in mice) or N-cadherin (in sheep). To determine whether the mitochondrial preparations were in a coupled state, mitochondrial respiration (oxygen consumption rates) was measured using a Seahorse XF24 Extracellular Flux Analyser (Seahorse Bioscience, Billerica, USA). Briefly, mitochondria (15 ug) were loaded into XF24 plates in 50 ul basal MAS (Seahorse Bioscience) buffer. Plates were subsequently centrifuged at 2500 g for 20 mins at 4 degrees. Mitochondria were then warmed by the addition of warmed (37°C) MAS buffer containing succinate (10 mM) and rotenone (2 uM). Basal respiration was established after the samples were allowed to equilibrate in the seahorse XF24, followed by injection of ADP (1 mM) to initiate State 3 respiration, then sequential injection of the ATP synthase inhibitor oligomycin, the proton ionophore carbonylcyanide p-trifluoromethoxyphenlhydrazone (FCCP) and the complex III inhibitor antimycin A, (all 1 µM) to allow for calculation of respiratory control ratios (state3/state 4 ratio). Mitochondrial membrane potential was also assessed to confirm the viability of isolated cardiac mitochondria using the fluorescent probe 5,5′, 6,6′,-tetrachloro-1,1,3,3′-tetraethylbenzimidazolylcarbocyanine iodide (1.5 µM JC-1, Molecular Probes, Eugene, OR, USA) [21] in the presence and absence of 2 µM FCCP and 2 µM antimycin A (respiratory chain inhibitor). Green and red fluorescence were measured at 485 nm/535 nm and 485 nm/590 nm (excitation/emission) respectively, and results expressed as a ratio of red/green raw fluorescence units (RFU).

### Mitochondrial [^3^H]-L-Arginine Uptake

Cardiac mitochondrial L-arginine transport was characterised by treating isolated mitochondria with various mitochondrial inhibitors as well as inhibitors or competitors of the y^+^ transport system, comprising L-lysine (10 mM), NEM (200 uM) and antimycin (2 uM). Mitochondrial pellets were re-suspended in uptake buffer (220 mM sucrose, 5 mM MgCl_2,_ 20 mM KCl, 10 mM KH_2_PO_4_, 10 mM HEPES/KOH, 10 mM sodium succinate, pH 7.4) and L-Arginine uptake was measured using radiolabeled [^3^H]-L-Arginine (specific activity 58 Ci/mmol, Perkin Elmer Life Sciences, Boston, MA, USA). Solutions containing 100 nM [^3^H]-L-arginine and increasing concentrations of unlabelled L-arginine (0.75–500 µM) were prepared in uptake buffer and incubated for 15 minutes at 37°C. Pellets were washed then resuspended in 0.5% SDS and measurement of radioactivity was made using liquid scintillation spectrometry. CAT1 mediated L-Arginine uptake was calculated as the difference between total uptake and 10 mM L-lysine sensitive uptake. Data is presented as nmol arginine uptake/mg protein.

### CAT-1 protein expression in isolated cardiac mitochondria

Mitochondrial protein (20–30 µg) was subjected to SDS-PAGE analysis using 7.5% acrylamide gels. After transfer, membranes were blocked for 1 hour at room temperature in 5% w/v skim milk, then incubated with CAT-1 antibody (1∶1000 in 5% w/v BSA, 1X TBS, 0.1% Tween20, polyclonal rabbit anti-CAT-1 custom made by Rockland Immunochemicals, Gilbertsville, PA, USA) overnight at 4°C. Signal detection was performed by chemiluminescence. Quantitative densitometric analysis was performed (Bio-Rad laboratories) and CAT-1 protein expression levels were indexed to 70 kDa subunit of mitochondrial complex II.

### Measurement of nitric oxide (NO) generation by isolated mitochondria and 3-nitrotyrosine as a marker of peroxynitrite formation

NO production in isolated mitochondria (20 µg) from healthy and failing hearts was measured in triplicate using 5 µM 4-amino-5-methylamino-2′,7′-dichlorofluorescein diacetate (DAF-FM, Molecular Probes, Eugene, OR, USA) as previously described [16]. Evidence of nitrosative stress was determined by measuring 3-nitrotyrosine levels in mitochondria isolated from healthy and failing hearts using a OxiSelect Nitrotyrosine competitive ELISA kit (Cell Biolabs Inc; San Diego, CA, USA).

### Mitochondrial CAT-1 overexpression in cardiomyocytes

Neonatal rat ventricular cardiomyocytes (NVCMs) were used for all cellular studies. NVMCs from D1-3 neonatal rats were isolated by enzymatic digestion as previously described [16]. Full length mouse CAT-1 was cloned into a vector (pPhi-yellowFP-mito, Evrogen, Moscow, Russia) containing a mitochondrial targeting sequence (MTS) to generate pCAT1-PhiYFP-MTS. CAT1-PhiYFP-MTS or PhiYFP-MTS were then cloned into the Adeno-Associated Virus (AAV) Vector pAAV-CMV-MCS-SV40pA-deltahPLAP to generate AAV6-CAT1-PhiYFP-MTS or AAV6-PhiYFP-MTS. Transfected cells were then subjected to various mitochondrial stresses including hypoxia/reoxygenation (3 hours hypoxia followed by 2 hours normoxia) [16] or exposure to the mitochondrial respiratory chain inhibitor, antimycin A (0.5 µM) for 5 hours. ROS production, mitochondrial membrane potential, mitochondrial respiration and ATP turnover along with lactate dehydrogenase release were then measured. ROS production was measured with 10 µM 2′,7′-dichlorodihydrofluorescein diacetate (H_2_DCF-DA, Molecular Probes) and mitochondrial membrane potential using the fluorescent probe JC-1 both as previously described [16]. Mitochondrial localisation of CAT-1 within cardiomyocytes was investigated by confocal microscopy with 50 nM MitoTracker Red (Molecular Probes Eugene, OR, USA).

Mitochondrial function was also examined in cells transfected with CAT1-YFP-MTS or YFP-MTS AAV following hypoxia/reoxygenation using the Seahorse XF24 Extracellular Flux Analyser. Briefly, NVCMs were plated into 24well Seahorse V7 plates at 6×10^4^ cells/well, as described above. Basal energetics were then established through the simultaneous measurement of oxygen consumption rate (OCR) and extracellular acidification rate (ECAR) in the Seahorse XF24, followed by sequential injection of oligomycin, FCCP and antimycin A (all 1 µM) to allow for calculation of basal respiration, OCR attributable to ATP turnover, uncoupled respiration and maximal and spare respiratory capacity. Mitochondria were also isolated from NVCMs as previously described [22]. Studies were performed in triplicate over at least three independent experiments.

### Statistical analysis

Data was analysed with by either Student t-tests or by one-way analysis of variance (ANOVA) followed by Bonferonni post-hoc tests for multiple comparisons. All statistical tests were performed using GraphPad Prism program, version 6 (GraphPad Software, San Diego, USA), and p<0.05 was considered indicative of statistical significance. All data are presented as mean ± SEM.

## Results

### Mitochondrial CAT-1 expression in healthy and failing hearts

**Figure 1:**
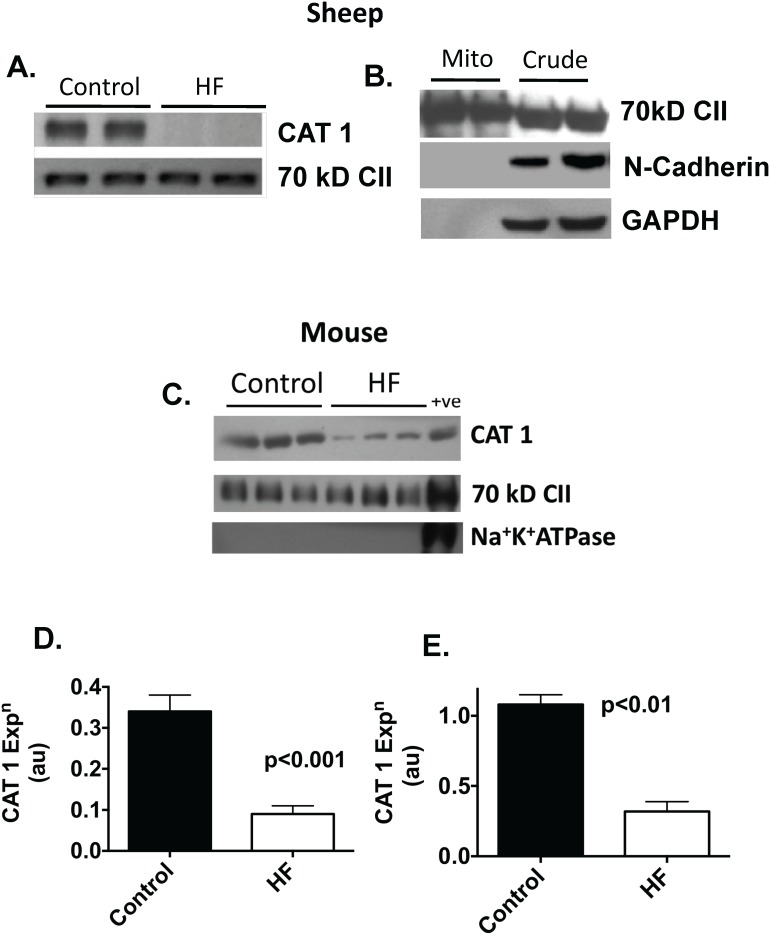
Western blot demonstrating reduced CAT1 expression in failing sheep mitochondria, normalized to expression of the 70(A). Western blot confirming the absence of contaminating sarcolemmal (N-cadherin) or cytosolic (GAPDH) proteins in mitochondrial preparation (**B**). Western blots showing reduced CAT1 in failing mouse mitochondria (**C**). Sarcolemmal Na^+^K^+^ATPase is absent from the mitochondrial preparation compared to crude homogenate (+ve). Bar graphs showing CAT1 expression in sheep (**D**) and mice (**E**) (n = 4 per gp).

To investigate the hypothesis that a specific L-arginine transporter is present within cardiac mitochondria, we conducted western blotting on mitochondrial samples to determine the expression of CAT-1. As shown in Figure 1A–C, CAT-1 was readily detected in mitochondria isolated from both sheep and mice. The absence of contaminating sarcolemma was confirmed by the absence of Na^+^K^+^ATPase in mice and by N-cadherin in sheep. In mitochondrial samples isolated from failing hearts there was a significant reduction in CAT-1 abundance compared with controls in both sheep (73%, p<0.001) and mice (70%, p<0.01) with heart failure (Figure 1A–E).

**Figure 2:**
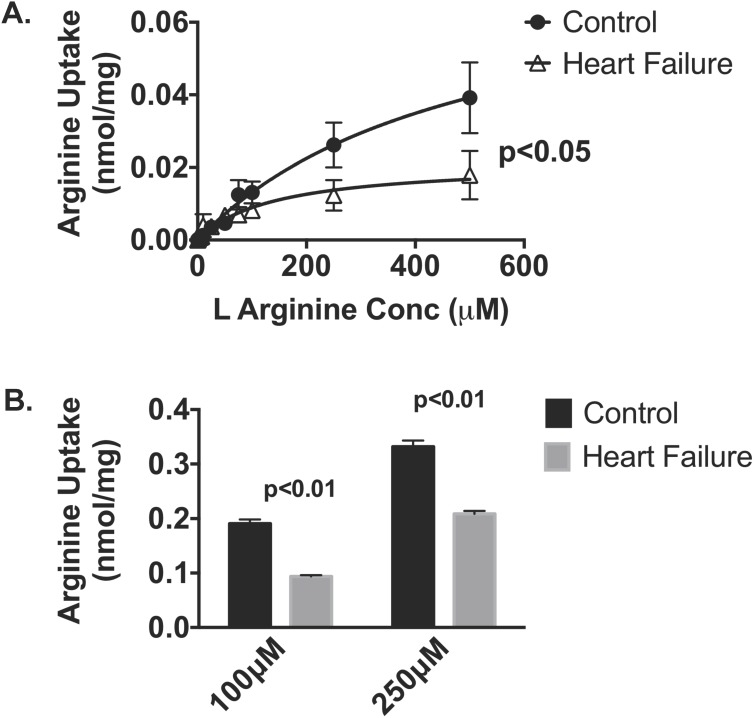
L-Arginine uptake was assessed in healthy and heart failure cardiac mitochondria from A. sheep (n = 5–7 per gp) and B. mice (n = 4 per gp) by measuring [^3^H]-L-arginine influx (15 mins incubation time) in the presence of varying concentrations of unlabeled L-arginine in. Data is presented as mean ± SEM.

**Figure 3:**
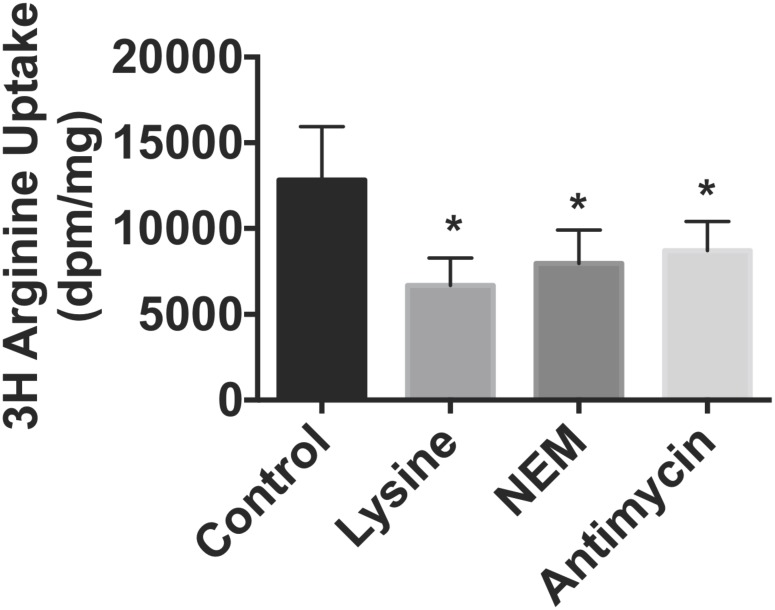
Mitochondrial [^3^H]-L-arginine uptake was measured in the presence of L-lysine (10 mM), NEM (200 µM) or antimycin (2 µM) (n = 5–7 per gp). Data is presented as mean ± SEM. *p<0.05 *vs.* control.

To corroborate the demonstration of CAT1 mitochondrial protein, we next evaluated the uptake of [^3^H] L-arginine in both models of HF. Preliminary L-arginine uptake time course studies over 30 minutes in isolated cardiac mitochondria demonstrated a progressive linear accumulation of [^3^H] L-arginine over time ranges from 1–25 minutes (R^2^ = 0.9905 data not shown) and accordingly an incubation time of 15 minutes was selected. In parallel we investigated mitochondrial respiration using the Seahorse platform. Mitochondrial viability was first confirmed by the presence of respiratory control ratios of ≥4. State 3 respiration, as assessed in the presence of succinate, was however significantly (p<0.05) decreased in mitochondria isolated from failing hearts compared to controls (997±117 *vs* 1726±198 pmol O_2_/min). As shown in Figure 2, there was a concentration dependent increase in L-arginine uptake in mitochondria isolated from the normal heart. By contrast, L-arginine uptake was significantly reduced in mitochondria from failing sheep and mouse hearts when compared with healthy controls. L-arginine transport by sheep mitochondria from control animals was significantly reduced by the competitive inhibitor L-lysine (48%, p<0.05), NEM (37%, p<0.05) and antimycin (32%, p<0.05) as shown in Figure 3.

### Nitric oxide production and nitrosative damage in HF mitochondria

To evaluate whether altered CAT1 expression and L-arginine transport was associated with an alteration in nitric oxide (NO) production we evaluated mitochondrial NO release fluorometrically. Under basal conditions, by DAF fluorescence, NO generation was modestly but significantly reduced in failing sheep mitochondria and compared to control (4404±797 vs 4004±852 rfu, p<0.05). In association, protein nitrosation, measured as 3-nitrotyrosine, was significantly (p<0.05) higher in mitochondria isolated from failing hearts (327±70 nM) versus mitochondria from healthy controls (109±31 nM).

### Effect of mitochondrial L-arginine availability during cardiomyocyte stress

**Figure 4:**
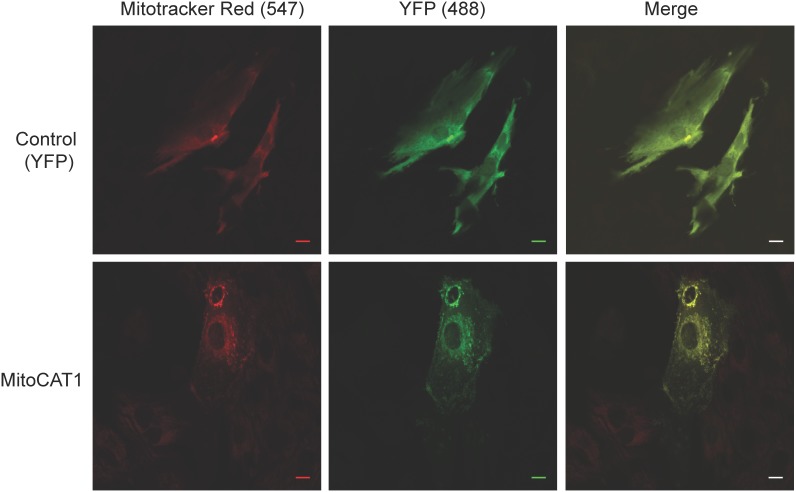
Representative fluorescent confocal images of cardiomyocytes (NVCMs) transfected with control YFP-MTS AAV6 (top panels) or CAT1-YFP-MTS AAV6 (lower panels) incubated with Mitotracker Red. Images confirm mitochondrial targeting in CAT1-YFP-MTS AAV6 transfected cardiomyocytes. Scale bar = 10 µm.

To extend the above observations we next aimed to investigate the potential role of mitochondrial L-arginine transport in the myocardial response to metabolic stress. To address this question we generated an adeno-associated virus (AAV) encoding CAT1-YFP-MTS that incorporates a mitochondrial targeting sequence allowing for CAT-1 overexpression specifically in the mitochondria and transfected isolated ventricular cardiomyocytes. Control transfections were performed using AAV-YFP-MTS. Expression and localisation of CAT-1 was confirmed using confocal microscopy with MitoTracker Red as shown in Figure 4.

To confirm that mitochondria from cardiomyocytes transfected with mitochondrial overexpression of CAT1 possessed an increased capacity for arginine uptake and to generate NO from arginine, we isolated mitochondria from CAT1-YFP-MTS and YFP-MTS AAV transfected cardiomyocytes. Mitochondria isolated from CAT1-YFP-MTS transfected cardiomyocytes had significantly greater capacity for ^3^H arginine transport compared to mitochondria from YFP-MTS transfected myocytes (1645±211 vs 772±254 dpm/mg protein, p = 0.03, n = 5). In conjunction, we determined the ability of 100 uM arginine to yield NO release as assessed by DAF fluorescence. In CAT1 transfected mitochondria, arginine caused a 1.72±0.16 vs 1.28±0.06 (p<0.05) fold rise in NO production.

**Figure 5:**
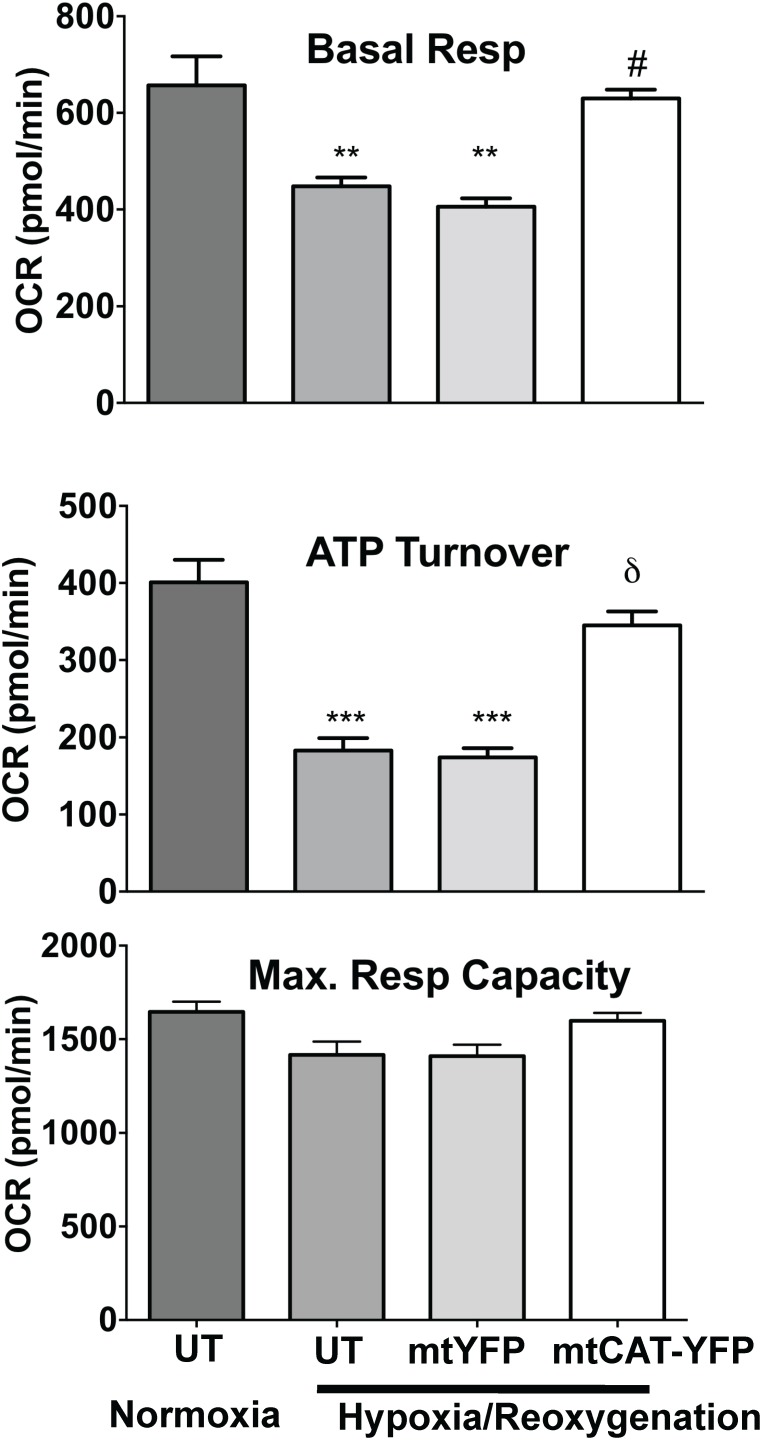
Bar graphs show oxygen consumption rates (OCR) due to basal respiration (top panel), OCR required for ATP turnover (mid panel) and maximal respiratory capacity (lower panel) as evaluated by oxygen consumption rates in isolated cardiomyocytes following hypoxia-reoxygenation. Cells were control untransfected (UT) or transfected with CAT1-YFP-MTS or YFP-MTS AAV6 for 72 hr then subjected to 3 hr hypoxia-2 hr reoxygenation. Data is presented as mean ± SEM. **p<0.01 and ***p<0.001 *vs*. non-transfected normoxic controls; δ p<0.05 and #p<0.01 *vs*. non-transfected hypoxia-reoxygenated NVCMs.

Basal respiration (Fig. 5A) was significantly decreased in NVCMs following hypoxia-reoxygenation (447±18 *versus* 657±60 pmol O_2_/min in normoxic cells, p<0.01). By contrast, NVCMs transfected with CAT1-YFP-MTS AAV were completely protected and exhibit significantly improved basal OCR compared with non-transfected cells following hypoxia-reoxygenation (630±18 pmol O_2_/min, p<0.05 versus non-transfected hypoxia-reoxygenation). Hypoxia-reoxygenation significantly decreased oxidative ATP turnover (p<0.001 *versus* normoxic controls, Fig. 5B), which was also significantly restored in CAT1-YFP-MTS AAV NVCMs (p<0.01 compared with non-transfected hypoxia-reoxygenated NVCMs, Fig. 5B). There were no significant changes in maximal respiratory capacity following hypoxia-reoxygenation (Fig. 5C). There were no significant differences in OCR or ATP turnover after hypoxia-reoxygenation in NVCMs transfected with the control AAV (YFP-MTS AAV) compared with non-transfected hypoxia-reoxygenated NVCMs (Fig. 5). CAT1-YFP-MTS transfection had no effect on basal respiratory rate (631±53 pmol O_2_/min), ATP turnover (361±37 pmol O_2_/min) or maximal respiratory capacity (1680±84 pmol O_2_/min) in normoxic NVCM.

Exposure of NVCMs to hypoxia with reoxygenation significantly reduced mitochondrial membrane potential (60±1.4% of normoxic controls, p<0.001) and significantly increased ROS production (144±2.3% of normoxic controls, p<0.001) as shown in Table 1. NVCMs treated with antimycin A also exhibited significantly decreased mitochondrial membrane potential (68±1.5% of untreated controls, p<0.001) and significantly increased ROS generation (142±4% of untreated controls, p<0.001, Table 1). In conjunction, LDH release, a marker of cell death or necrosis, significantly increased during both hypoxia-reoxygenation and with antimycin A treatment (200±8 and 255±23 *versus* control, 7±0.2 arbitrary units x10^3^, both p<0.001, Table 1).

**Table 1:** Effect of mitochondrial L-arginine availability on isolated ventricular cardiomyocytes during mitochondrial stress.

	Control	Antimycin A	CAT1-MTS AAV +Antimycin A	YFP-MTS AAV+Antimycin A	Hypoxia-Reox^n^	CAT1-MTS AAV+Hypoxia- Reox^n^	YFP-MTS AAV+Hypoxia- Reox^n^
**ROS production (% control)**	100	142±4***	120±1^†^**	155±1.1***	144±2.3***	121±1.6^#^**	146±2.4***
**Mitochondrial membrane** **potential (% control)**	100	68±1.5***	86±1.1^††^**	65±1.8***	60±1.4***	79±1^§^**	57±1.7***
**LDH release** **(absorbance units×10^3^)**	7±0.2	.255±22***	172±5^†^***	291±9***	200±8***	123±5^§^***	203±5***

NVCMs were either untreated, or infected with either CAT1-YFP-MTS AAV6 or YFP-MTS AAV6. Cells were then subjected to 3 hr hypoxia-2 hr reoxygenation or treated with antimycin A for 5 hr. ROS generation and mitochondrial membrane potential were measured in these cells using the fluorescent probes H_2_DCF-DA and JC-1 respectively. LDH release was also measured as a marker of cell death (n = 3 individual experiments with 4 replicates per experiment for fluorescent probes, or n = 3 for LDH). Data represents mean ± SEM (*p<0.05, **p<0.01 and ***p<0.001 *vs*. normoxic untreated controls; ^†^p<0.01 and ^††^p<0.001 *vs*. antimycin A; and ^#^p<0.01 and ^§^p<0.001 *vs*. untreated cells after 3 hr hypoxia-2 hr reoxygenation).

By contrast, NVCMs transfected with CAT1-YFP-MTS AAV exhibit significantly reduced ROS generation and significantly improved mitochondrial membrane potentials following both hypoxia-reoxygenation or antimycin A treatment when compared with their non-transfected controls (Table 1). ROS production during hypoxia-reoxygenation was limited to 121±1.6% in CAT1-YFP-MTS AAV NVCMs *versus* 144±2.3% in non-transfected cells (p<0.01), whilst mitochondrial membrane potential depolarized to 79±1% in CAT1-YFP-MTS AAV cells compared with 60±1.4% in non-transfected cells (p<0.001). ROS production following antimycin A treatment was significantly lower in CAT1-YFP-MTS AAV NVCMs than in non-transfected cells (120±1% *versus* 142±4%, p<0.01) and mitochondrial membrane potential was significantly higher in CAT1-YFP-MTS AAV cells (86±1.1% compared with 68±1.5% in non-transfected controls, p<0.001). Furthermore, CAT1-YFP-MTS NVCMs had significantly decreased LDH release following hypoxia-reoxygenation and antimycin A treatment (p<0.001 and p<0.01 respectively compared with non-transfected control, Table 1). There were no significant differences in ROS generation, mitochondrial membrane potential or LDH release after 2 hrs reoxygenation or with antimycin A treatment in NVCMs transfected with the control AAV (YFP-MTS AAV) compared with relevant non-transfected cells (Table 1). There was also no significant difference in LDH release in normoxic cells transfected with either CAT1-YFP-MTS or YFP-MTS AAV when compared with non-transfected normoxic controls.

## Discussion

In the present study we aimed to determine whether mitochondria possess a specific transport system for the translocation of L-arginine from the cytoplasm and to investigate its role in the pathophysiology of cardiovascular disease states associated with heightened oxidative stress, specifically heart failure and hypoxia-reoxygenation stress. Our study was predicated upon emerging evidence that NO plays an important role in the regulation of several mitochondrial processes and that mitochondria may contain enzymatic systems including NOS which generate NO [10].

We demonstrated evidence for the concentration dependent accumulation of L-arginine in the mitochondria of both mice and sheep. The plasmalemmal transport of L-arginine in many cell types including cardiomyocytes is predominantly mediated by the biochemical system y^+^ carrier, which is principally represented by the CAT-1 transporter [23]. The presence of such a transporter in the mitochondria has previously remained unclear in mammalian cells. Further evaluation by western blotting on isolated cardiac mitochondria and confocal microscopy of isolated cardiomyocytes confirmed the presence of CAT-1 in mitochondria, thus providing new insights into the mechanism responsible for the transport of L-arginine into mitochondria. Previously studies have reported the presence of a mitochondrial ornithine transporter in yeast, which exhibited some capacity for L-arginine transport with a relatively low Km [24]. Additionally a mitochondrial arginase has also been described [25], together suggesting the presence of a complex system for the regulation of intra-mitochondrial L-arginine content. In our study we showed that the mitochondrial transport of L-arginine was significantly reduced by the cationic amino acid L-lysine consistent with a y^+^ system mediated transport system. The present study did not investigate the precise localization of the mitochondrial CAT1 transporter. Furthermore we did not establish the relationship between transporter kinetics and the influence of various mitochondrial substrates.

Having established the presence of a mitochondrial CAT-1 L-arginine, we aimed to determine whether mitochondrial CAT1 expression was altered in heart failure. We found that both mitochondrial L-arginine uptake and mitochondrial expression of the CAT-1 L-arginine transporter were significantly reduced in the failing heart, in both models of heart failure. These data are consistent with our previous findings that depressed vascular and myocardial L-arginine transport plays a key role in the pathophysiology of a range of cardiovascular disorders such as hypertension and heart failure [15], [26], [27]. In the context of heart failure, most previous studies of arginine transport have been conducted in relation to the concomitant decrease in endothelial function, by virtue of its influence on NO production[26]. Similarly, it has been proposed that diminished endothelial arginine transport could contribute to the impairment of endothelial function in hypertension [28], [29]. Mechanistically, previous studies of CAT1 activity and expression demonstrated negative effects of angiotensin II and particularly protein kinase C, of which both have pathophysiologic relevance to heart failure [30]. In the context of the present study, a concomitant decrease in plasmalemmal arginine transport would be expected to further magnify the effects of a decrease in mitochondrial arginine transport on mitochondrial function. In relation to the present study, we did not specifically study the mechanism responsible for the regulation of mitochondrial CAT1 expression.

In the current study we also found that mitochondria isolated from failing hearts have significantly lower NO production and significantly higher levels of 3-nitrotyrosine, indicative of increased oxidative/nitrosative stress. 3-nitrotyrosine, a widely used marker of oxidative and nitrosative damage in human and animal disease states, is formed when peroxynitrite attacks tyrosine residues in proteins. Peroxynitrite is known to cause irreversible inhibition of many mitochondrial components including the respiratory chain (specifically complex I and cytochrome c oxidase), the Kreb’s cycle enzyme aconitase and Mn-superoxide dismutase [31].

To further investigate the notion that altered mitochondrial L-arginine transport may influence the overall cellular response to oxidative stress we examined whether augmented mitochondrial L-arginine would provide cellular protection during hypoxia-reoxygenation stress. Ventricular cardiomyocytes with mitochondrial overexpression of CAT1 showed a significant decrease in ROS production, perseveration of mitochondrial membrane potential together with evidence of significantly less cellular injury following either hypoxia-reoxygenation or antimycin A exposure. Furthermore, the deleterious effects of hypoxia-reoxygenation on mitochondrial respiration and ATP turnover were attenuated by enhanced mitochondrial CAT1 expression. Although we did not examine the effects of increased mitochondrial CAT1 expression in an experimental model of heart failure, we hypothesise that it would be beneficial given that heart failure is associated with elevated oxidative stress and that mitochondrial arginine transport is reduced as demonstrated above.

Our findings of a cellular protective effect of increased mitochondrial L-arginine transport and NO production are entirely consistent with the findings of Chouchani and colleagues who demonstrated that S-nitrosation of complex I, using a mitochondrial selective NO donor, provided cardioprotection by reducing oxidative damage [7]. Similarly, S-nitrosation of complex I by nitrite has also been shown to be cardioprotective [32]. As such, S-nitrosation of key cysteine residues could protective effects during ischemic injury by preventing the irreversible effects of exposure to peroxynitrite. For example, peroxynitrite can cause direct permeabilisation of mitochondrial membranes leading to the formation of mitochondrial permeability transition pore (MPTP) with subsequent inhibition of ATP synthesis which can lead to cellular necrosis [33]. High concentrations of NO may transiently modify mitochondrial oxygen consumption by interacting with cytochrome *c* oxidase [34]. Nitric oxide has also been reported to exert other aspects of mitochondrial function including the prevention of mitochondrial calcium accumulation**,** activating expression of a number of mitochondrial proteins including PGC-1α, increasing the production of mitochondrial DNA or inhibiting the pathway of apoptosis [34]–[37]. Other studies suggest that reduced mitochondrial NO availability impairs mitochondrial biogenesis and decreased myocardial NO levels induce a switch from fatty acid metabolism to glycolytic metabolism [38].

In the present study we did not aim to specifically determine the source of mitochondrial NO. The presence of a constitutively expressed mitochondrial NOS enzyme has been proposed based on the demonstration of mitochondrial NO generation using a range of techniques including electroparamagnetic spin trapping, radiometry, chemiluminescence and fluorescence microscopy [10], however this remains controversial. A mitochondrial NOS has been proposed to be a component of the inner membrane and which generates NO in a calcium-dependent manner [39]–[46]. The identity of a putative mitochondrial NOS also remains speculative, with some evidence for a neuronal isoform NOS based upon proteomic analysis of mitochondrial extracts and the absence of mitochondrial NO production in nNOS knockout mice [47], [48]. The mechanism by which a putative mtNOS localises to the mitochondria is unknown, but may include post-translational modifications or interactions with other proteins [35]. Whilst our observations are consistent with a paradigm in which the mitochondrial L-arginine transporter plays a key role in providing substrate to a mitochondrial NOS which in turn generates NO to regulate mitochondrial function and to provide protection during cellular stress, it is possible that the mitochondrial L-arginine transporter influences mitochondrial function in other ways. For example the generation of ornithine via arginase could participate in other mitochondrial bioenergetic activities.

### Conclusion

Our study provides the first evidence for the presence of a CAT-1 isoform L-arginine transporter in mammalian cardiac mitochondria. We demonstrated a significant reduction in L-arginine uptake and NO production in mitochondria obtained from the failing heart and this was accompanied by evidence of nitrosative modification of the mitochondrial membrane. Increasing mitochondrial L-arginine availability by transfection of a mitochondrially targeted CAT-1 significantly improved cardiomyocyte responses to mitochondrial stresses, reducing oxidative stress and improving survival. Taken together, these data provide new insights into the nature of myocardial mitochondrial L-arginine transport, its role in mitochondrial biology and the pathogenesis of cardiovascular disease.
